# Getting Your Peaks in Line: A Review of Alignment Methods for NMR Spectral Data

**DOI:** 10.3390/metabo3020259

**Published:** 2013-04-15

**Authors:** Trung Nghia Vu, Kris Laukens

**Affiliations:** 1 Department of Mathematics and Computer Science, University of Antwerp, Middelheimlaan 1, Antwerp, B-2020, Belgium; E-Mail: Kris.Laukens@ua.ac.be; 2 Biomedical Informatics Research Center Antwerp (biomina), Wilrijkstraat 10, Edegem, B-2650, Belgium

**Keywords:** NMR, alignment, preprocessing, peak shifts, Nuclear Magnetic Resonance

## Abstract

One of the most significant challenges in the comparative analysis of Nuclear Magnetic Resonance (NMR) metabolome profiles is the occurrence of shifts between peaks across different spectra, for example caused by fluctuations in pH, temperature, instrument factors and ion content. Proper alignment of spectral peaks is therefore often a crucial preprocessing step prior to downstream quantitative analysis. Various alignment methods have been developed specifically for this purpose. Other methods were originally developed to align other data types (GC, LC, SELDI-MS, *etc.*), but can also be applied to NMR data. This review discusses the available methods, as well as related problems such as reference determination or the evaluation of alignment quality. We present a generic alignment framework that allows for comparison and classification of different alignment approaches according to their algorithmic principles, and we discuss their performance.

## 1. Introduction

Although Nuclear Magnetic Resonance (NMR) spectroscopy is a powerful analytical tool for quantitative metabolomics profiling, one of the aspects that hamper robust differential analysis is the fact that the resonance frequencies of peaks can undergo shifts. A variety of factors, often related to an imperfect control of experimental conditions, contribute to inconsistent peak shifts, including physicochemical interactions and differences in pH, temperature, background matrix or ionic strength [1,2,3]. Some of these effects can be (partially) avoided by using adjusted sample preparation protocols, for example by buffering samples to avoid pH-induced chemical shifts [1,3]. On the other hand, there is a clear need for computational approaches to correctly align corresponding peaks across spectra. If peaks are inconsistently shifted across different spectra, they will not be properly matched and downstream univariate or multivariate quantitative analysis of their signal intensities can be compromised.

**Figure 1:**
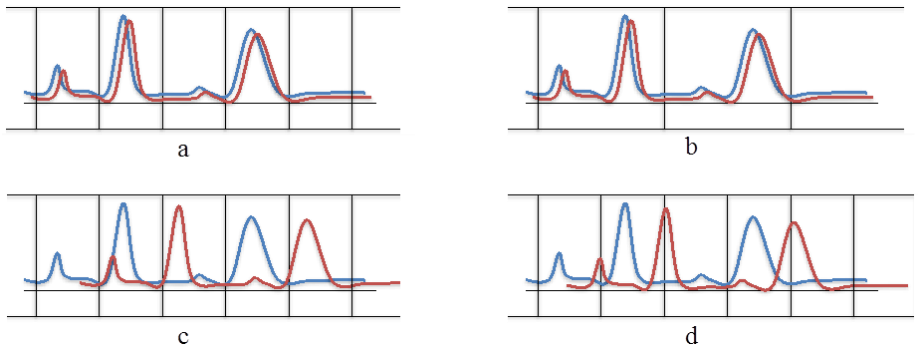
Hypothetical examples of how binning addresses peak shifts. (**a**) A good binning—each peak corresponding between the red and blue spectrum end up the same bin. (**b**) Multiple peaks end up in a single bin. (**c**) Incorrect matching of overlapping peaks—the first peak of the red spectrum falls in the same bin as the second peak of the blue spectrum. (**d**) Peak shifts across the boundaries of bins.

A simple and popular solution to extract intensities from multiple spectra prior to comparative analysis is spectral bucketing or binning. Binning consists of dividing the spectra into small buckets (typically 0.04 ppm), which are ideally large enough to encompass peak shift variations [1,3]. The intensity of each bucket is subsequently calculated from the area under the curve. Traditional binning overcomes small peak-shifts, and reduces data complexity (Figure 1a). This however comes at a price, as it leads to drastic reduction of data resolution. If multiple peaks end up in the same bucket, information is lost (Figure 1b). Furthermore, strong shifts may lead to non-corresponding peaks incorrectly ending up in the same bin (Figure 1c). Shifts that exceed the boundaries of a bin, will be reflected in integrated bin intensities (Figure 1d). After binning, the statistical analysis is carried out on the extracted bin intensities, and peaks are assigned to metabolites. It is clear that above-mentioned weaknesses can lead to poor metabolite quantitation.

To deal with these problems, various improvements to the binning approach have been developed. For example, instead of using fixed bin sizes, Davis *et al.* [4] introduced an adaptive binning that creates flexible bin sizes based on the peaks detected in the reference spectrum. An improvement of adaptive binning is Adaptive intelligent binning [5] which reduces the required user interventions. Anderson *et al.* [6] also applied kernel-based methods for binning and demonstrated that the proposed Gaussian binning is more robust than traditional binning. These methods require a certain degree of user expertise [7]. Recently, Sousa *et al.* [7] released a less complicated method, the optimized bucketing algorithm (OBA), in which bin sizes are optimized by setting their boundaries at the local minima of the average spectrum. Binning methods are using widely since they are easy to use and show acceptable performances. However, binning does not easily handle larger NMR peak shifts.

The solution to process and compare spectra with peak shifts consists of peak alignment. A number of peak alignment approaches have been specifically developed for NMR spectroscopy. Other methods were originally proposed for similar data, such as LCMS or GCMS spectra. In this review we discuss and compare the available peak alignment methods that are directly and without special adaptation applicable to NMR spectra.

## 2. A Generic NMR Spectrum Alignment Framework

**Figure 2:**
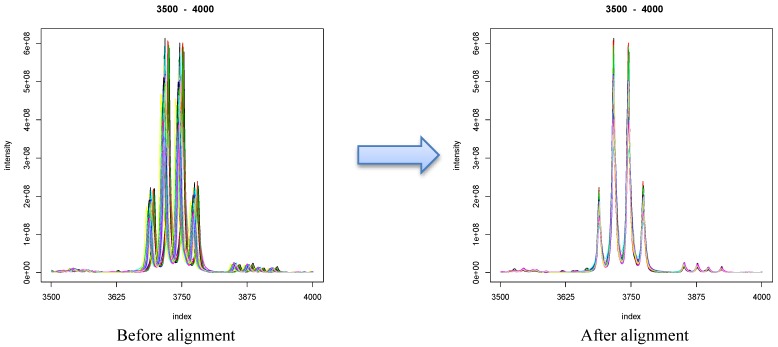
Example of a spectral region before and after alignment using CluPA [8]. The example comes from the Wine dataset [9,10].

NMR spectrum alignment is a process to correct for variations in the position of peaks across NMR spectra, by introducing a series of shifts that individual data points undergo. The process is illustrated in Figure 2.

**Figure 3:**
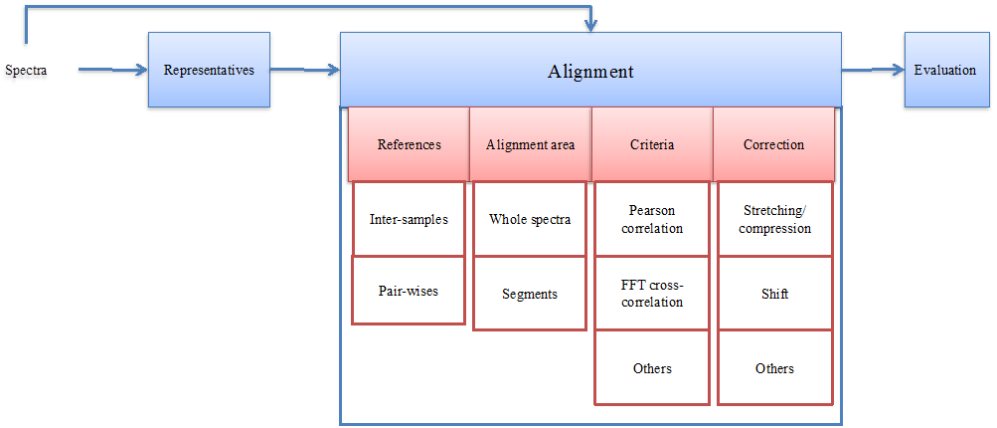
A general framework of Nuclear Magnetic Resonance (NMR) spectrum alignment methods. The stacked blocks with white background represent possible methodological variations.

The calculation of the optimal set of shifts to align spectra is computationally non-trivial, and a number of choices need to be made along this process, each with repercussions on the final outcome. A list of NMR alignment methods is presented in Table 1. Most methods fit in the general alignment framework presented in Figure 3. In the next sections, we discuss each step of this framework, as well as the methodological choices that are to be made.

## 3. Working on Extracted Peaks Instead of Full Spectra

While most NMR alignment methods work directly on the data points of the spectra, some approaches work with representatives of the spectra instead, by first converting spectra into peak lists. The size of such a list of peaks extracted or “picked” from the spectrum is much smaller than the original spectrum. This improves computational performance of the subsequent alignment, or allows for computationally more demanding techniques to be used.

**Table 1:** List of methods and their features.

Short Name	Full Name	Reference	Technique	Target Function	Peak Picking?	Number of Parameters	Original Applied Data	Segment-Wise?	Pair-Wise?	Correction Method	Software
PLF	Partial Linear Fit	[11]	Segmentation model by consecutive peaks distances less than window size D	Sum of squared differences in intensity	No	2 (window *D* and shift *S*)	1D NMR	Yes	Yes	Shift	NA
COW	Correlation Optimized Warping	[12]	Dynamic programming	Pearson correlation coefficient	No	2 (*m*: length of segments and *t*: slack or the max. allowable shift)	Chromatograpic data	yes	Yes	Insert and deletion	(1)
PAGA	Peak alignment by genetic algorithn	[13]	Genetic Algorithm	Pearson correlation coefficient	No	6 - Based on GA (normalize geometric ranking *q*=0.8, population size, number of generations, segment size, max. allowable shift, linear interpolation *ra*)	1D NMR	Yes	Yes	Shift & Insert and deletion	NA
PARS	Peak alignment using Reduced Set	[14]	Breadth first search (BFS), Dynamic Programming (DP), complexity reduced dynamic programming (crDP)	Euclidean distances	Yes	2 (search window size, mismatch weight)	1D NMR, Gas Chromatography	No	Yes	Shift	(+)
DTW	Dynamic Time Warping	[15]	Dynamic programming	Squared Euclidean distance	No	2 (*T*(*x*,*y*) local continuity constraint; *x* = largest block distance covered by any of the rules, *y* = max. number of horizontal / vertical consecutive transition allowed for)	Chromatograpic data	No	Yes	Insert and deletion	(1)
PABS	Peak alignment by Beam search	[16]	Beam search algorithm	Pearson correlation coefficient	No	3 (ranges of segment number, sideway movement and interpolation)	1D NMR	Yes	Yes	Shift & Insert and deletion	(+)
PAPCA (*)	Peak alignment by PCA	[17]	Principle Component Analysis	CORREL	No	1 (correlation threshold 0.8)	1D NMR	No	No	Shift	(+)
PTW	Parametric Time Warping	[18]	Global polynominal model	Root mean squared (RMS)	No	1 (degree of polynomial warping function)	Chromatograpic data	No	Yes	Polynominal model	(2)
PAFFT	Peak alignment by FFT	[19]	FFT + segmentation model by equal size segments	FFT cross-correlation	No	2 (segment size: *segsize*, max. allowable shift)	Chromatograpic data	Yes	Yes	Shift	(3)
RAFFT	Recursive alignment by FFT	[19]	FFT + Recursive segmentation model from global to local	FFT cross-correlation	No	1 (max. allowable shift)	Chromatograpic data	Yes	Yes	Shift	(3)
SpecAlign	NA	[20]	Sliding windows	Minimal matched peak distances	No	1 (window size *w*)	Mass Spectrometry	No	Yes	Insert and deletion	(3)
FW	Fuzzy Warping	[21]	Fuzzy logic for matching most intense peaks	Maximize fuzzy membership Gaussian function	Yes	1 (the number of most intense peaks)	1D NMR	No	Yes	Insert and deletion	(4)
GFHT	Generlized Fuzzy Hought Transform	[22]	Hough transform	Hough score	Yes	3 (expansion factor alpha, step size, lower vote threshold)	1D NMR	No	No	NA	NA
RSPA	Recursive segment-wise peak alignment	[23]	Recursive segmentation model	FFT cross-correlation	Yes	6 (peak height threshold, splitting threshold, min. segment size, validation of segment alignment, max. allowable shift, alignment acceptance)	1D NMR	Yes	Yes	Shift & Insert and deletion	(+)
PCANS	Progressive Consensus Alignment of NMR Spectra	[24]	Segmentation model+Dynamic programming + progressive consensus alignment	Scoring by similarity between peaks calculated by height, half height and position of peaks	Yes	5 *(minScoreN, minScoreD,* gap penalty, boundary penalty, max. allowable shift *maxCS)*	1D NMR	Yes	No	Shift	(5)
BAA (*)	Bayesian approach for alignment	[25]	Bayesian modeling	Bayesian estimation	No	3 (noise variance, two parameter values in diagonal entries of diagonal covariance matrix)	1D NMR	No	Yes	Polynomial model	NA
icoshift	interval correlation shifting	[10]	Segmentation model by equal size segments or manually selecting segments	FFT cross-correlation	No	2 (the number of intervals or the length of interval *l*, max. allowable shift)	1D NMR	Yes	Yes	Shift & Insert and deletion	(6)
CluPA	hierarchial Cluster-based Peak Alignment	[8]	Segmentation model by hierarchical clustering	FFT cross-correlation	Yes	1 (max. allowable shift)	1D NMR	Yes	Yes	Shift	(7)

(*): This name is not from the authors, but assigned by us for convenience; NA: The software implementation information is not available; (+): The implementation of the algorithm can be requested from the authors; (1): http://www.models.life.ku.dk/DTW_COW/; (2): http://cran.r-project.org/web/packages/ptw/index.html/**;** (3): http://powcs.med.unsw.edu.au/research/adult-cancer-program/services-resources/specalign/**;** (4): http://code.google.com/p/automics/ refer to [26]**;** (5): http://gomezlab.bme.unc.edu/tools/**;** (6): http://www.models.life.ku.dk/icoshift/**;** (7): http://code.google.com/p/speaq/

Many effective and advanced peak picking algorithms are available. In all cases, accurate peak detection is required to build a quality alignment. For a discussion and comparison of peak picking methods we refer the reader elsewhere [27]. Below we will focus on how the extracted peak lists are used by different alignment algorithms.

In general, peak lists are used to compute how individual data points of each spectrum should be shifted to optimally align all input spectra. First, the extracted peak lists of different spectra are compared to find corresponding peaks. To align these peaks, a set of shifts is computed, which are subsequently applied to the intact spectra. Methods differ in how they find corresponding peaks and their regions, in how shifts are computed, and in how they are applied.

The first example is PARS [14]. Extracted peaks of a reference and the sample spectra are first matched using search algorithms on distance maps of derived peak lists. The derived shift that corresponding peaks need to undergo is subsequently used to align the sample spectra against the reference.

In FW [21], feature vectors are created from the most intense peaks of reference and sample spectra. Through fuzzy warping, it then establishes the correspondence between the most intense peaks of the sample spectra. The alignment is done through piecewise interpolation of the sample spectrum to the corresponding regions of the reference spectrum.

PCANS [24] uses a “Naive Alignment scheme” to match highly similar peaks between peak lists of spectrum pairs. Then it builds the corresponding segments from the matches. Finally, it undergoes another alignment on the segments following a dynamic programming scheme.

RSPA [23] and CluPA [8] both use peak lists to find the shifts needed to align corresponding segments, in a recursive scheme from global (the entire spectrum) to local (a small subsection of the spectrum), in order to go from a crude to a more refined alignment. RSPA merges consecutive peaks to build segments and finds the corresponding segments based on their proximity defined by the closest center positions. CluPA uses hierarchical clustering on peak list of reference and sample spectra to find the corresponding segments.

GFHT [22] also uses peak detection approaches, but the detected peaks are used in a different way. The approach does not work directly with the peak lists to align corresponding peaks, but applies image-processing techniques on the entire dataset to detect corresponding peaks via the spectral peak patterns.

The major advantage of an intermediate peak-picking step is the reduced data size. Consequently, these methods are generally faster than methods working on whole spectra, like COW [12] or DTW [15].

## 4. Alignment with or without a Reference Spectrum

A second criterion by which we can classify alignment approaches is the fact whether a reference spectrum is needed or not. In pairwise methods, a reference spectrum is selected to which all the other spectra are subsequently aligned. With inter-sample methods, all samples are taken into account for the alignment.

### 4.1. Pairwise Methods, Based on a Selected Reference Spectrum

Most NMR alignment methods are based on pairwise approaches, which are generally less complex. In pairwise methods, a reference spectrum is selected or created first. Other spectra are aligned to this reference one by one. The reference spectrum should be representative for the whole dataset and ideally contains all peaks of interest. Due to its strong impact on the ultimate alignment, a number of reference selection approaches have been proposed. There are generally two reference types. Either the reference is a virtual spectrum that is artificially created from the dataset, or the reference is a directly selected spectrum from the dataset.

A virtual reference spectrum can be built in different ways. The reference spectrum may be a median or average spectrum constructed from the dataset [10,11]. In DTW [15], a virtual spectrum is recalculated from the first PCA loading vectors, resulting from a PCA analysis of the untreated data. The virtual spectrum can be used directly as a reference against which the other spectra are aligned. However, artifacts introduced during the creation of a virtual spectrum may lead to distorted peaks after alignment. A solution consists of selecting from the experimental spectra as a reference the spectrum that is most similar to the virtual spectrum, rather than using the virtual spectrum itself. For example, Skov *et al.* [28] proposed to use the spectrum that is most similar to the loading of the first principle component in a PCA model of the untreated data.

Alternatively, users can select the reference from multiple trials. In FW [21], several reference candidates are selected, according to the mean value of the correlation coefficient of individual spectra with all the remaining spectra. A higher value indicates a better reference. The final reference is selected after evaluation of the alignment. More simply, a user can sometimes manually select the reference [14]. The reference may also be selected as the spectrum with the highest (Pearson) correlation to other spectra, under the assumption that this will yield the most representative reference and best alignment to all experimental spectra. Skov and coauthors [28] recommended using the spectrum that has the largest similarity index, which is defined as the product of the correlation coefficients to the other spectra. Veselkov *et al.* [23] modified the similarity index of Skov *et al.* [28] to avoid a dominant influence of large peaks on the correlation coefficient values, by scaling local areas to equal variance prior to computing the correlation coefficient. Vu *et al.* [8] selected the reference based on a goodness value, which summarizes how close its peaks are to the corresponding peaks of all other spectra. This method also allows users to manually set the reference to specific segments, since a single spectrum may not be the best reference for all segments. MacKinnon *et al.* [29] automated this by dividing the length of the spectra into *m* global segments and assigning as a reference for each segment the one having the largest similarity to the others.

Even though reference-based approaches are relatively simple and popular, there are some disadvantages. Due to the variability between spectra not all important peaks are present in all individual spectra and thus in the selected reference. Significant differences may exist between spectra depending on the group they belong to. The quality of the results therefore depends on the selected reference spectrum.

### 4.2. Inter-Sample Methods, without Using a Reference Spectrum

Although most alignment methods follow pairwise approaches that depend on a reference, there are a few that can do alignment without a reference.

PAPCA [17] detects peak regions of whole spectra in which peak shifts occur and then aligns the regions by shifting. It derives orthogonal principle components by applying PCA on the whole spectral data. Then it slides the first derivatives of a variety of simulated peak-shapes along the second component. The correlation coefficient between the first derivatives and the underlying part of the second component at each frequency point is calculated. The spectral regions of interest (SROI) are selected for alignment if the points in the regions show high correlation.

By considering the whole spectral data as an image, GFHT [22] finds the shift-pattern based on an image processing technique called generalized fuzzy Hough transform. The shift pattern is the inter-sample peak position of a peak. First, peak detection is applied on the spectra and an indicator matrix is constructed. The model peak is selected for the whole spectra. To model the peak shifts, the shift pattern is multiplied by an expansion parameter. The Hough is then iterated through the parameter. Meanwhile, it records the Hough scores (the values indicate the fitting of the current shift pattern to the peak shifts) into a matrix *H*. The procedure starts with the global maximum in *H*, assigns peak identity, iterates the local maximum in *H* and stops when all peaks are extracted. An improved version of GFHT [30] incorporates a multicomponent peak shift model (MCSM) by using PCA to deal with more complex data. The advantage of generalized fuzzy Hough transform methods is their capacity of dealing with the fact that the spatial order of peaks in the spectra can change, or in other words that the positions of peaks in a given spectrum are reversed in other spectra.

A third inter-sample method is PCANS [24], which avoids selecting a reference spectrum by repeatedly creating consensus spectra through integration of pair-wise spectrum comparisons until a final consensus spectrum remains. The final output includes the set of input spectra aligned to the final consensus spectrum.

## 5. Alignment of Whole Spectra or Alignment of Spectrum Segments?

The next distinction between different alignment workflows can be made according to whether they align complete spectra or smaller segments.

A first group of methods, considers the whole spectra for alignment. For example PTW [18] and BAA [25] both present each spectrum as a function of data points, build the models of the shifts between two spectra and minimize the difference between the spectra. FW [21] uses Fuzzy warping to find maximally corresponding peaks in whole spectra, and then uses an interpolation function for alignment. GFHT [22] considers whole spectra as a 2-dimensional image and aligns based on the Hough transform. DWT [15] uses a dynamic programming algorithm to warp two spectra. It builds a warping path to match points from the reference with the sample spectra. This method has the disadvantage that the peak shapes of aligned spectra are easily distorted due to artifacts. In an improvement, VPdtw [31] uses a variable penalty in the Dynamic Time Warping process. SpecAlign [20] uses a sliding window to move from point to point and aligns by insertion and deletion.

These methods usually get slow when the size of the spectra increases. To address this performance problem, a class of methods was developed to divide spectra into smaller corresponding segments, to which the alignment is subsequently applied. PLF [11] uses a window size *D* to separate adjacent segments. PAFFT [19] and icoshift [10] divide the spectra into equal segments (or allow to manually select segments, in the case of icoshift) and align each segment. COW [12] divides spectra into equal segments used for alignment but it compresses or stretches (insertion and deletion) the segments instead of aligning them separately. PAGA [13] and PABS [16] use search algorithms such as genetic algorithm and beam search to determine the division points of segments. CluPA [8], RSPA [23] and RAFFT [19] find the corresponding segments by recursive strategies from global to local to refine the alignment. Doing alignment on segments instead of on the whole spectra significantly speeds up the computational time.

## 6. Criteria or Target Function

Alignment is an optimization problem, in which a set of parameters needs to be estimated. A typical factor in optimization techniques is the “target function”, which is the criterion by which candidate or partial solutions are evaluated throughout the alignment process. Even though different NMR alignment methods have different underlying principles, they often use similar optimization criteria. A common criterion is Pearson correlation coefficient, which can be maximized between segment pairs [12,13,16]. Intuitively, well-aligned spectra should have a high Pearson correlation. This coefficient is also commonly used for the evaluation of a completed alignment, as discussed later. Other methods use distances between the spectra. PARS [14] uses Euclidean distance, DTW [15] uses squared Euclidean distance, and vpDTW [31], which is an improved version of DTW, uses *L*1 norm, *i.e.* the sum of the absolute differences between data points in the spectra. Other criteria are derived specifically from the underlying algorithms. In PAPCA [17], a PCA algorithm is applied to all the spectra. It finds the regions that maximize the correlation CORREL that is created from the information of the simulated first derivatives along the second principle component and the underlying part of the second principle component. GFHT [22] uses a fuzzy membership Gaussian function as optimization criterion. More recently, because of their high speed, some groups started using FFT cross-correlation as the criterion for segment alignment. This was first introduced by Wong *et al.* in PAFFT and RAFFT [19], and was later used effectively by others [8,10,23].

## 7. Correction Methods

After finding the corresponding points or segments in spectra, the alignment methods need to correct the misalignment. A first class of methods uses stretching/compression (or insertion/deletion) to correct the misalignment spectra. Usually, stretching/compression is done by a linear interpolation to fit the corresponding segments in the reference [12,15,21]. Alternatively, it can be done by least squares quadratic polynomial fit as in SpecAlign [20]. Stretching/compression may lead to information loss and can also cause artifacts in the spectra. A second, widely used way to correct for the misalignment is shifting [8,14,17,19,24]. Shifting just moves a segment to the left or right for several data points, to match the reference spectrum. Shifting also leads to some information loss, and some new lines are added to the spectra. Ideally, the points that are selected to shift are at the baseline of the spectra or are the lowest intensity point, in order to avoid distorted peak shapes. Instead of adding lines to fill gaps, an interpolation function can be used. Nevertheless, artifacts remain hard to avoid. Some methods combine shift and interpolation to smooth the spectra [10,13,16,23]. Besides stretching/compression and shifting, a third group of methods uses a polynomial model for correction. Eilers *et al.* [18] and Kim *et al.* [25] directly estimate the aligned spectra from their warping functions through polynomial models.

## 8. Alignment Assessment and Evaluation

After alignment, the aligned spectra need to be evaluated to assess the quality of alignment methods. Below we discuss different levels of evaluation of aligned spectrum sets.

### 8.1. Visualization

**Figure 4:**
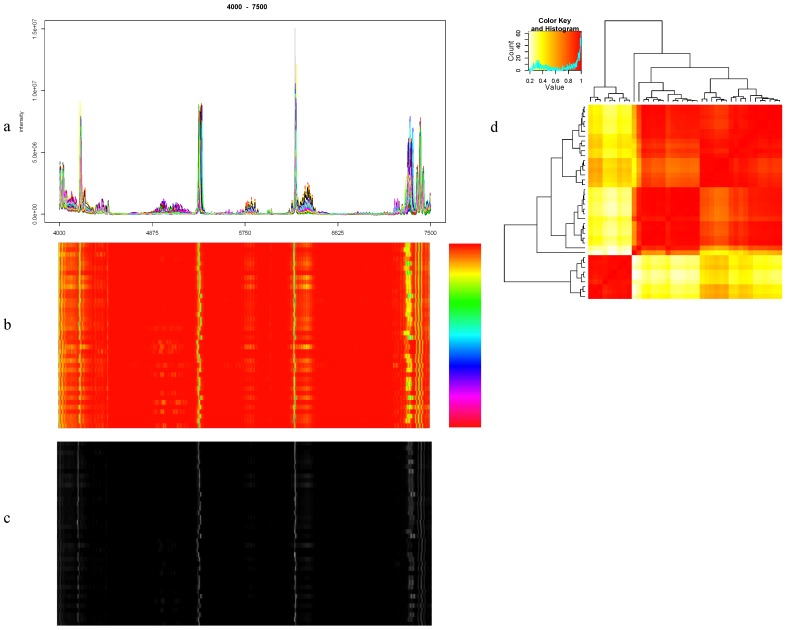
Examples of evaluation by using visualizations for a region in the Wine data [9,10]. (**a**) Spectral plot. (**b**) Spectra image. (**c**) Grey scale plot. (**d**) The heatmap of spectra correlation.

Visualization is a powerful approach to rapidly assess the properties of a dataset, and in the context of this review, to evaluate the quality of an alignment procedure. We can visualize a number of relevant features in a few different ways, as illustrated in Figure 4. A simple visualization are spectral plots [10,13,16,19,20,21,23,25] in which whole spectra or a region are overlaid and plotted to see how the spectra were aligned (Figure 4a). A related and effective approach consists of presenting all spectra in a single image of size *N*x*M*, where *N* is the number of spectra and *M* is the number of data points in each spectrum. The color of each pixel reflects the intensity of a given peak in a given spectrum as in GFHT [22] (Figure 4b). Somewhat less information-rich are the equivalent grey scale representations [8,18,23], where the intensity of each pixel (from grey to white) corresponds to peak intensity (from low to high) (Figure 4c). High intensity peaks prominently show up in these visualizations, and the effects of the alignment can thus be evaluated. Alternatively, a heatmap can be constructed from the correlation coefficient matrix [8,21,23]. Correlations between all sample pairs are computed to create the matrix. The matrix is then plotted as an image in which the color of each pixel represents the degree of correlation between spectrum pairs.

### 8.2. Quantitation of Similarity between Spectra

A good alignment usually leads to an increased correspondence between spectra. Inter-spectrum similarity is thus a useful criterion for the evaluation of alignments. The most popular approach to evaluate inter-spectrum similarity consists of comparing average Pearson correlation coefficients of spectra before and after alignment [2,8,12,13,16,21,22,23,25]. The Pearson correlation coefficient can also be combined with a Wallis filter [28] to avoid the fact that the correlation is mainly affected by the highest peaks while the low peaks are almost ignored. Kim et al, 2010 [25] compared the average root mean squares of spectra from different alignment methods. A smaller value then indicates a better alignment. Torgrip *et al.* [14] evaluated the correlation of spectra using the inner product of the first derivatives of the two data vectors scaled to unit norm, because this value is sensitive to differences in peak location and peak shape. They also introduced an evaluation using the peak match score “PMS” to measure the number of corresponding peaks and also the presence or absence of corresponding peaks in samples. Alternatively, the average peak shifts [19] can be used, which are the average shifts of several top intense peaks. Lower average peak shifts indicate a better alignment.

### 8.3. PCA Analysis

Principle Component Analysis (PCA) is a technique to project high-dimensional data into linearly uncorrelated vectors or principle components, in such a way that the first component represents the majority of the variance in the data, with subsequent components representing decreasing variance. PCA is a natural way to express data and discover data patterns based on their similarities. The fact that features of PCA before and after alignment are different can be used for evaluation of alignment in several ways. Vogels *et al.* [11] introduced a ratio inter-intra distance that is the average Euclidean distance for *p* principle components of samples to their group mean divided by the average distance between groups. Higher ratio values indicate a better group separation. The variance of principle components can also be used for evaluation [14,17,21,22,23,24] based on the principle that after alignment, the variance of the first principle component should increase while the variance of the second PC decreases. Several methods [10,13,15,21] based the evaluation upon the fact that after alignment, the PCA scoring plots typically present more clearly the group separation or trend of the data. Forshed *et al.* [13] proposed an evaluation method using the linear combination of loadings from PCA that describes the orthogonal direction from a line separating the two groups in the scoring plots. One of its strengths is the fact that it reveals the spectral information (the distinct peaks) that separates two groups.

### 8.4. Classification Model Analysis

In studies where metabolome profiles are used to compare or classify different sample classes, the classification accuracy itself gives an indication on alignment quality. A good alignment should improve the accuracy. In general, any classifier that is used for classifying metabolome profiles can be used for this purpose, for example SVM [32,33], Random Forests [34] and PLS-DA [35]. For a detailed description of classification techniques that are applicable to metabolome data we refer to relevant reviews [3,36].

Instead of using classifiers as a black box, we can also evaluate alignment according to specific properties derived from a classification model. For example, a back-scaled loading coefficients of an OPLS classifier has been used [24,37]. The loadings coefficients are positive (negative) if the spectral features are higher (lower) in control (treated) groups. Other authors used a PLS model [2,10,11,21,22] and used performance metrics such as the root-mean square error of cross validation (RMSCV) for evaluation. Forshed *et al.* [13,38] measured the distance between groups from their approximated distributions generated from two scoring vectors of PCA and PLS-DA which show good group separation. A higher value reflects a better separation between classes and thus a better alignment.

### 8.5. Other Evaluation Approaches

There are a number of other metrics to evaluate alignment quality. One is the relative standard deviation of peak intensity as in GFHT [22]. After alignment, the variance between spectra should be very low. Wu *et al.* [21] use hierarchical clustering, under the assumption that alignment should lead to improved clustering and spectra from the same groups should cluster together. After alignment, Statistical Total Correlation Spectroscopy (STOCSY) analysis [23,39,40] should improve identification and determination of structural and biological correlations. An example of STOCSY can be found in RSPA [23]. To evaluate the alignment, Skov *et al.,* [28] proposed a simplicity value, a peak factor and the warping effect (the combination of the two other parameters). The simplicity value is based on the properties of the singular value composition (SVD). In unaligned data less total variation is explained by the first few singular values then in aligned data. The peak factor indicates how much the total spectra have changed, and is derived from normalizing the difference between Euclidean norms of aligned and unaligned spectra. Giskeødegård *et al.* [2] and MacKinnon *et al.* [29] also used these approaches for evaluation. 

## 9. Method Complexities

### 9.1. Computational Complexity

Comparing the speed of all NMR alignment methods is not trivial, since the computational time of some algorithms depends on parameter setting. For example, searching in PABS [16] is faster than in PAGA [13], but both heuristic algorithms are stopped according to user-defined stop criteria. If they stop too early, the global search optimum may not be reached. Some implementations are not freely available. Therefore, we rely on published results [2,39,41] to try to collect information for computational time comparison.

SpecAlign [20] and PLF [11] can be considered to be the fastest since their time is linear to the number of data points in a spectrum. Peak-picking approach-based methods such as RSPA [23] and CluPA [8] take additional time for the peak detection process. They are generally a bit slower than non-peak-picking methods such as icoshift [10], PAFFT [19] and RAFFT [19], which use the same FFT cross-correlation method for finding the shift steps. Recently, Giskeødegård *et al.* [2] made a detailed comparison of five popular NMR alignment methods, listed according to speed (from high to low) as follows: icoshift [10] > vpDTW [31] > PABS [16] > PTW [18] > COW [12]. Furthermore methods that use dynamic programming, like COW [12] and DTW [15], are more computationally demanding. Even though COW [12] is considered faster than DTW [15], it was reported to take minutes to several hours for high-resolution NMR spectra (~10000 up to 64000 data points) [2,10,23] on a personal computer. The faster methods listed above typically require seconds to minutes to complete, depending on the dataset size. For several other methods we have no performance information.

### 9.2. Usage Complexity (Method Meta-Parameters)

Most NMR alignment methods rely on a set of user-defined parameters. Optimizing these parameters is a challenge for most users. Different data sets may require different parameter settings. In practice, most users try a few parameter sets and select the set that yields the best result, without a guarantee that they selected the best possible set of parameters. The more parameters a method requires, the more complicated and difficult it becomes to use. Consequently, some methods attempt to reduce the number of user-set parameter without sacrificing (as much as possible) the performance of the alignment. An overview of the numbers of parameters of several algorithms is presented in Table 1. Peak-picking based methods additionally require setting the parameters of the peak detection step, which is outside the scope of this discussion and omitted from the table.

## 10. Alignment of 2D NMR Data

1D ^1^H-NMR is used in the majority of NMR-based metabolome profiling studies because it is a fast approach to determine the biomolecular constituents of a sample. When working on complex biological samples however, there is often significant overlap between different signals. 2D NMR, usually in the combination ^1^H-^13^C, is a good alternative to overcome this problem. 2D NMR improves the understanding of the structure of an organic compound, but it is also affected by peak shifting problems. Since 1D NMR alignment methods cannot be applied directly on 2D NMR data, dedicated 2D NMR alignment methods are needed. Only a few methods are available for this type of data. Binning can be used to compare imperfectly aligned 2D NMR datasets, but has the disadvantages discussed earlier for 1D NMR. Since 2D NMR datasets can be considered as images, image-processing techniques from the computer vision field could be applied for finding matching points in the image. However, applying them to high-resolution 2D NMR data remains a challenge. Zheng *et al.* [42] proposed a heuristic algorithm and a similarity measure to maximize an objective function that captures the alignment quality. Recently, Robinette *et al.* [43] proposed a hierarchical strategy for 2D NMR alignment.

## 11. Conclusion and Future Work

NMR spectrum alignment remains a difficult problem for which there is no golden standard solution. For example the problem of peak order changes mentioned by Csenki *et al.* [22] cannot easily be solved, as most alignment methods are restricted by the assumption of similar peak order between spectra. Furthermore the time complexities and in particular the number of parameters that need to be optimized remain significant problems in which there is room for improvement. Nevertheless, the extensive list of available methods for NMR spectrum alignment addressed in this review offers metabolome researchers a powerful toolbox to extract the maximum out any dataset for which peak shifts are a practical issue.
